# A *C. elegans* Model for Mitochondrial Fatty Acid Synthase II: The Longevity-Associated Gene W09H1.5/*mecr-1* Encodes a 2-*trans*-Enoyl-Thioester Reductase

**DOI:** 10.1371/journal.pone.0007791

**Published:** 2009-11-16

**Authors:** Aner Gurvitz

**Affiliations:** Section of Physiology of Lipid Metabolism, Institute of Physiology, Center for Physiology, Pathophysiology and Immunology, Medical University of Vienna, Vienna, Austria; University of Florida, United States of America

## Abstract

Our recognition of the mitochondria as being important sites of fatty acid biosynthesis is continuously unfolding, especially in light of new data becoming available on compromised fatty acid synthase type 2 (FASII) in mammals. For example, perturbed regulation of murine *17β-HSD8* encoding a component of the mitochondrial FASII enzyme 3-oxoacyl-thioester reductase is implicated in polycystic kidney disease. In addition, over-expression in mice of the *Mecr* gene coding for 2-*trans*-enoyl-thioester reductase, also of mitochondrial FASII, leads to impaired heart function. However, mouse knockouts for mitochondrial FASII have hitherto not been reported and, hence, there is a need to develop alternate metazoan models such as nematodes or fruit flies. Here, the identification of *Caenorhabditis elegans* W09H1.5/MECR-1 as a 2-*trans*-enoyl-thioester reductase of mitochondrial FASII is reported. To identify MECR-1, *Saccharomyces cerevisiae etr1*Δ mutant cells were employed that are devoid of mitochondrial 2-*trans*-enoyl-thioester reductase Etr1p. These yeast mutants fail to synthesize sufficient levels of lipoic acid or form cytochrome complexes, and cannot respire or grow on non-fermentable carbon sources. A mutant yeast strain ectopically expressing nematode *mecr-1* was shown to contain reductase activity and resemble the self-complemented mutant strain for these phenotype characteristics. Since MECR-1 was not intentionally targeted for compartmentalization using a yeast mitochondrial leader sequence, this inferred that the protein represented a physiologically functional mitochondrial 2-*trans*-enoyl-thioester reductase. In accordance with published findings, RNAi-mediated knockdown of *mecr-1* in *C. elegans* resulted in life span extension, presumably due to mitochondrial dysfunction. Moreover, old *mecr-1(RNAi)* worms had better internal organ appearance and were more mobile than control worms, indicating a reduced physiological age. This is the first report on RNAi work dedicated specifically to curtailing mitochondrial FASII in metazoans. The availability of affected survivors will help to position *C. elegans* as an excellent model for future pursuits in the emerging field of mitochondrial FASII research.

## Introduction

In comparison to the long-established research area dedicated to studying cytosolic fatty acid synthase type 1 (FASI), the field of mitochondrial FASII in eukaryotic cells is only just beginning to emerge. Nevertheless, significant advances have been made recently in determining the protein components of mitochondrial FASII in both yeast [1], [2], [3], [4], [5], [6] and mammals [7], [8], [9], [10], [11], [12]. These studies have provided a clearer picture of how FASII contributes to lipoic acid synthesis and subsequent enzyme lipoylation [13], and also established a probable link to RNA processing [14] and human disease [12], [15]. Moreover, protozoan FASII has been exposed as a vital process that occurs in the apicoplast of *Plasmodium falciparum* and *Toxoplasma gondii* [16], and data on FASII in *Trypanosome brucei* underscored the importance of this pathway to mitochondrial function [17], [18], [19]. However, delays have been encountered in generating mouse knockout models, and therefore there remain gaps in the information that urgently need bridging. These open issues relate not only to the specific role played by FASII with respect to RNA processing and the identity of mitochondrially produced fatty acids other than lipoic acid, but importantly also to the consequence to animals of curtailing mitochondrial FASII altogether.

Eukaryotic cells undertake *de novo* acyl carrier protein (ACP)-dependent biosynthesis of fatty acids via two methods: i) by way of a cytosolic process comprised of an associative FASI system, and ii) using a compartmentalized FASII design in which the enzyme activities reside on dissociated proteins. Although bacterial FASII has been extensively studied [20] and the existence of a plant plastid process has long been known [21], eukaryotes have generally been thought to contain only a cytosolic pathway. This view has since been refocused following the discovery of an additional mitochondrial FASII in mammals, protozoa, and fungi. In the yeast *Saccharomyces cerevisiae*, a functional FASII is critical for mitochondrial function and morphology, and deletion of FASII genes results in a respiratory growth phenotype characterized by underdeveloped mitochondria lacking assembled cytochromes, insufficient levels of lipoic acid and, in some cases, loss of mitochondrial DNA (reviewed in [22], [23]).

**Figure 1:**
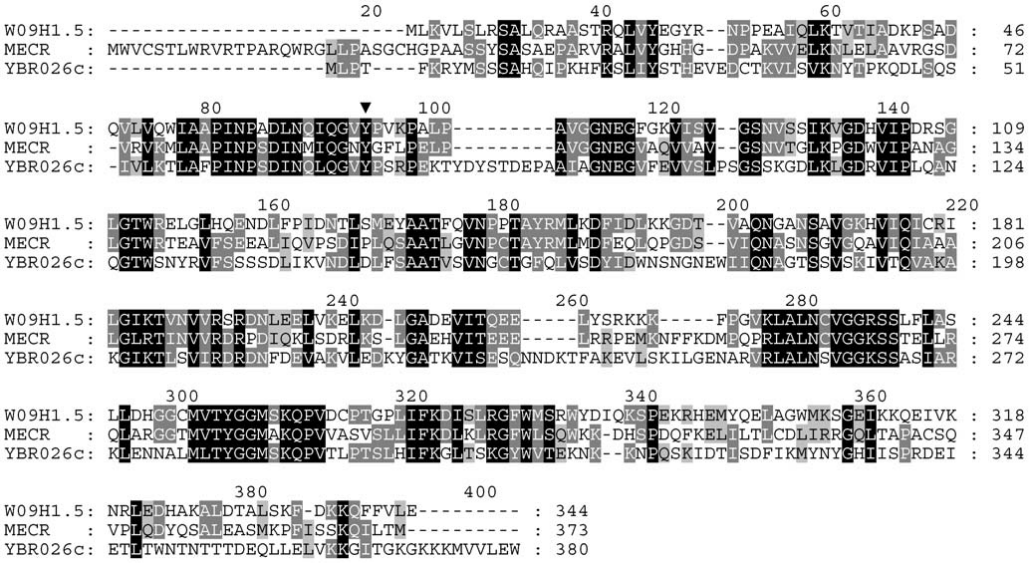
Similarity of *C. elegans* W09H1.5 to human MECR/NRBF-1 and yeast YBR026c/Etr1p. Comparison of the deduced amino acid sequences. Dashes indicate the arrangement of the sequences for best fit. Black shadings refer to conserved amino acid residues among the three sequences whereas the darker and lighter grey shadings denote regions with more relaxed residue similarities not necessarily shared by the full set of sequences. The inverted triangle points to a conserved catalytic tyrosine residue (Tyr-94) [10]. The Genbank accession numbers used were: W09H1.5, CAB04958.1; MECR, CAI14330.1; and YBR026c, NP_009582.1.

The requirement for a good animal model in which to undertake advanced studies on mitochondrial FASII might be satisfied by the nematode *Caenorhabditis elegans*. The *C. elegans* genome has been fully sequenced [24], and a cursory glance at the databases reveals that the nematode has a multitude of genes with the potential to encode FASII enzymes, including a physiologically functional 3-oxoacyl-ACP reductase [25]. The nematode genome also codes for several proteins (e.g. W09H1.5) that resemble yeast Etr1p and its corresponding human homologue MECR (Fig. 1). Such candidate *mecr-1* genes could be tested for function by undertaking complementation studies on a yeast *etr1*Δ mutant lacking mitochondrial 2-*trans*-enoyl-ACP reductase Etr1p [6]. The respiratory phenotype of *etr1*Δ mutant cells was shown previously to be rescuable by ectopically expressing known or candidate 2-*trans*-enoyl-ACP reductases, including *Escherichia coli* FabI [6], *Mycobacterium tuberculosis* InhA [26], *Candida tropicalis* Etr1p or Etr2p [6], [27], and *Homo sapiens* MECR/NRBF-1 [10].

As a first step in laying down the foundation for exploiting *C. elegans* as a model organism for FASII studies, the complete retinue of nematode FASII enzymes must be identified. Here, *S. cerevisiae* was employed as a heterologous platform for investigating whether *C. elegans* W09H1.5 represents a physiologically functional 2-*trans*-enoyl-ACP reductase. This was done by determining the ability of the nematode protein to supplant fungal Etr1p. Mutant *etr1*Δ cells expressing W09H1.5 were examined for 2-*trans*-enoyl-thioester reductase activity, respiratory growth on glycerol and lipoic acid production, and the findings are reported herein. Since a previous genome-wide screen identified W09H1.5 as one of ninety RNAi inactivations that extended nematode longevity [28], which suggested that FASII alterations may be meaningful in metazoans, inactivation of *C. elegans* W09H1.5 was also undertaken here so as to verify whether it was indeed required for normal mitochondrial function and aging. The identification of W09H1.5 as encoding an enzyme of compartmentalized fatty acid biosynthesis is discussed in terms of the utility of applying *C. elegans* as a model system for FASII studies as well as the importance of FASII for nematode life span extension due to reduced mitochondrial functions.

## Results

### *C. elegans* W09H1.5 restores respiratory growth to *S. cerevisiae etr1*Δ cells

The *C. elegans* genome (www.wormbase.org) contains at least two genes that are homologous to yeast mitochondrial Etr1p (Blast values and % lengths in brackets): W09H1.5 (3.9e–37, 81.7%) and Y48A6B.9 (4.5e–27, 63.6%). The conceptual amino acid sequences of these proteins were additionally analyzed using three algorithms (MitoProt, Psort II, and TargetP) which calculate the N-terminal region of a given protein that could support a mitochondrial leader sequence. MitoProt predicted (in brackets) that the polypeptide sequences relating to W09H1.5 (0.8937) and Y48A6B.9 (0.6487) had a probability of representing mitochondrial proteins that was higher than the known yeast mitochondrial protein Etr1p (0.6333). However, when using a further algorithm, PSORT II, W09H1.5 was clearly identified as a mitochondrial protein while Y48A6B.9 was determined to be cytoplasmic. Moreover, TargetP also supported the notion that W09H1.5 (0.887) was more likely to be mitochondrial than Y48A6B.9 (0.207). Since W09H1.5 (2.2e–72, 97.1%) is also more similar than Y48A6B.9 (6.9e–62, 95.7%) to the human homologue MECR/NRBF-1, the focus of the subsequent investigation was centered solely on W09H1.5.

**Figure 2:**
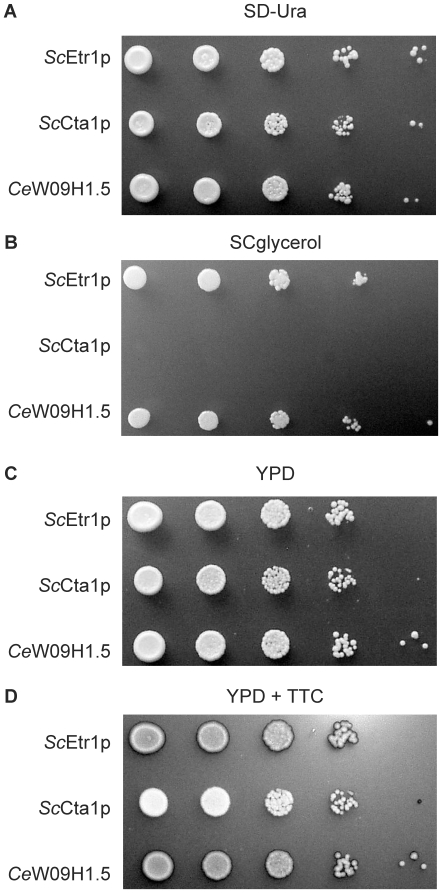
Phenotype rescue of a yeast *etr1*Δ mutant strain expressing nematode W09H1.5. *S. cerevisiae* BJ1991*etr1*Δ cells producing native mitochondrial *Sc*Etr1p, peroxisomal *Sc*Cta1p, or *C. elegans* MECR-1 (*Ce*W09H1.5), were grown overnight in SD-Ura medium that exerted selective pressure for plasmid presence. The yeast cultures were subjected to sequential tenfold dilution and spotted onto the indicated solid media. A and C. SD-Ura or YPD glucose media were used to verify whether dilution was applied similarly to all three strains. B. On SCglycerol medium, mutant yeast cells expressing the nematode protein were compared with the self-complemented positive control strain for respiratory growth, whereas cells enriched for fungal Cta1p that cannot grow on this non-fermentable carbon source were applied to the plate as a negative control. D. Cells grown on YPD medium that does not require respiration-competent mitochondria were overlaid with 2,4,5-triphenyltetrazolium chloride (TTC), which is metabolized rapidly by respiring cells to form the red chromophore, whereas even at higher densities fermenting cells remain white for a while.

To determine whether *C. elegans* W09H1.5 could represent a 2-*trans*-enoyl-ACP reductase, the corresponding gene was expressed behind the promoter of the yeast *CTA1* gene encoding peroxisomal catalase A (Cta1p), without resorting to an additional yeast mitochondrial leader sequence (MLS). *CTA1* transcription is only weakly derepressed on non-fermentable carbon sources such the glycerol used here for the complementation assays [29], [30]. The aforementioned yeast *etr1*Δ mutant cells lacking mitochondrial 2-*trans*-enoyl-thioester reductase activity were transformed to uracil prototrophy with *URA3*-marked plasmids expressing native Etr1p (positive control), fungal Cta1p (negative control), or *C. elegans* W09H1.5 (tester strain), and cultured overnight in liquid SD-Ura glucose medium. Following sequential tenfold dilution, cells were spotted onto SD-Ura or SCglycerol plates that were incubated at 30°C until single colonies appeared. The results in Fig. 2A showed that all three strains had returned to uracil prototrophy, indicating presence of the expression plasmids, whereas Fig. 2B demonstrated that like the situation with the self-complemented strain, mutant cells expressing W09H1.5 could grow on glycerol. Hence, the respiratory deficient phenotype of mutant *etr1*Δ cells could be rescued by the expression of *C. elegans* W09H1.5 to a similar extent as by native Etr1p, indicating functional complementation, while those expressing Cta1p could not proliferate on glycerol. Given the homology of W09H1.5 to Etr1p, and that W09H1.5 reversed the physiological defects of *etr1*Δ yeast cells without requiring an extra fungal MLS, W09H1.5 is likely to represent a mitochondrial 2-*trans*-enoyl-CoA/ACP reductase and its gene is hereafter referred to as *mecr-1*.

### Yeast *etr1*Δ cells expressing MECR-1 contain 2-*trans*-enoyl-thioester reductase activity

Preparations of soluble protein from BJ1991*etr1*Δ cells producing *C. elegans* MECR-1 were assayed for 2-*trans*-enoyl-thioester reductase activity. To stimulate elevated transcription and higher protein expression levels from the oleic acid-inducible *CTA1* promoter [29], [30], yeast cells were grown overnight in a medium that was comprised of this fatty acid [31]. Thereafter, the cells were collected by centrifugation, broken using glass beads, and soluble protein extracts were prepared. Enzyme assays conducted with 2-*trans*-hexenoyl-CoA (*trans*-C_6:1(2)_) as substrate were performed on soluble protein extracts obtained from positive control *etr1*Δ cells over-expressing native Etr1p, and this gave rise to an NADPH-dependent oxidation of the C_6_ substrate at the rate of 3.21 µmol/mg protein x min^−1^. Since any reductase activity potentially present in cells over-expressing Cta1p was below the detection limit of the assay used, this confirmed the specificity of the assay to detect only highly active reductases. When soluble protein extracts made from mutant cells over-expressing MECR-1 were applied to this assay, this resulted in an NADPH-dependent oxidation at the rate of 0.21±0.11 (S.D., n = 3) µmol/mg protein x min^−1^, demonstrating that nematode MECR-1 contained appreciable reductase activity.

### Nematode MECR-1 repairs the mitochondrial electron transfer chain in *etr1*Δ mutants

Previous work showed that deletion of the yeast *ETR1* gene results in mutant cells with defective respiration [32], but that this mutant phenotype is reversible when mutant cells are supplied with active compartmentalized reductases from a number of very different organisms [6], [10], [26], [27]. It could be anticipated, therefore, that in those *etr1*Δ mutants that had recovered the ability to could grow on glycerol as a consequence of expressing nematode MECR-1 (Fig. 2B), cytochrome complexes would assemble and the electron transport chain be regenerated. To demonstrate qualitatively that respiratory growth of mutant cells expressing yeast Etr1p or nematode MECR-1 coincided with a regenerated electron transfer chain, a method was employed in which an overlay comprising of 2,4,5-triphenyltetrazolium chloride (TTC) was applied to the aforementioned serially diluted cells that were left to grow for 4 d on rich-glucose YPD medium. TTC represents a redox indicator for monitoring cellular respiration, and quickly turns red when applied to respiring cells. The assay showed that mutant *etr1*Δ cells expressing Etr1p or MECR-1 were effective at metabolizing TTC, as evidenced by the rapid buildup of the red chromophore, whereas mutants enriched for peroxisomal Cta1p turned red only very slowly (Fig. 2C,D). The results confirmed that the restored ability of these *etr1*Δ cells to divide and grow on glycerol medium was due to their rehabilitated mitochondrial respiratory functions.

### Expression of MECR-1 restores lipoic acid production in the *etr1*Δ mutant strain

FASII supplies the C_8_ substrate for lipoic acid synthesis, whose latter deficiency in the *etr1*Δ mutant can be reversed when a *bona fide* reductase is provided ectopically [23]. The level of lipoic acid present in the extracted contents of the three yeast strains was measured in duplicates. The negative control yeast strain (*etr1*Δ cells enriched for Cta1p) gave rise to 14 ng lipoic acid per gram wet weight yeast cells, while mutant cells ectopically expressing fungal Etr1p produced 144 ng lipoic acid per gram wet weight. Since ectopic expression of MECR-1 in the yeast mutant gave rise to a wild-type level of 141 ng lipoic acid per gram wet weight, this provided additional supporting evidence that MECR-1 was functional within FASII.

### Depletion of MECR-1 in *C. elegans* protects against aging and increases life span

**Figure 3:**
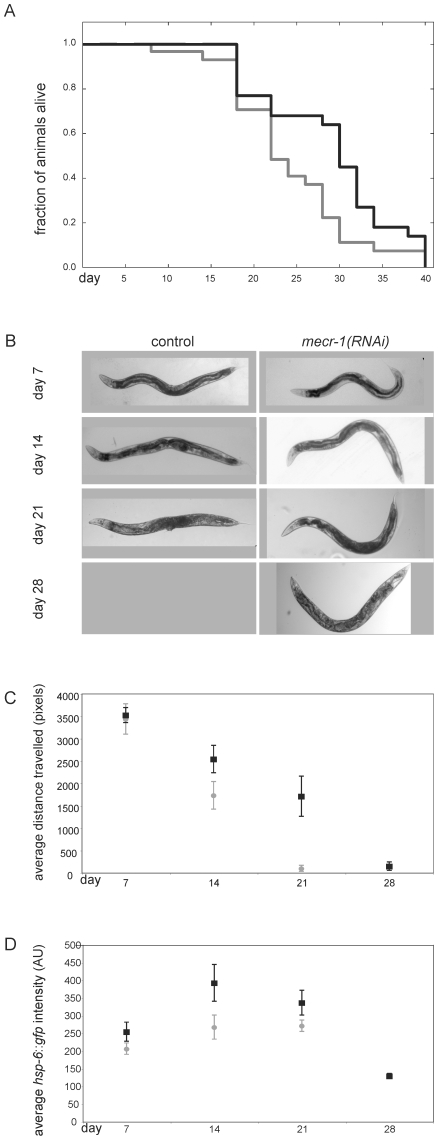
Depletion of MECR-1 in *C. elegans* slows aging. A. Longevity of control (gray) and *mecr-1(RNAi)* (black) *C. elegans*. B. Morphology of control and *mecr-1(RNAi) C. elegans* throughout life span. C. Motility of control (gray circles) and *mecr-1(RNAi)* (black squares) animals throughout life span. D. Induction of *hsp-6*::*gfp* in control (gray circles) and *mecr-1(RNAi)* (black squares) animals throughout life span. In B – D, the number of viable control worms was too small to allow an analysis.

A previous genome-wide RNAi screen suggested that W09H1.5 depletion results in increased longevity [28]. Hence, control and *mecr-1(RNAi)* animals were used here to determine the extent of life span increase. Animals were subjected to *mecr-1* RNAi from hatching onwards, and scored for viability every two days. The mean life span of control animals was 21.5±1 d while *mecr-1(RNAi)* animals lived on average 34% longer (mean life span 29±3, n =  four independent experiments). A representative viability curve is shown in Fig. 3A. Brood size and overall body size were not affected by MECR-1 depletion (data not shown). Additionally, *mecr-1(RNAi)* worms appeared generally healthier than control animals of the same age (Fig. 3B), which was most evident in their intestinal morphology. In order to improve the assessment of whether old *mecr-1(RNAi)* worms were more vigorous than control animals, crawling was measured throughout their lifetimes. Worms were chronically subjected to *mecr-1* RNAi and assayed for distance crawled in a given time period at weekly intervals for four weeks (Fig. 3C). Control and *mecr-1(RNAi)* worms had similar levels of activity at 7 d. Thereafter, control worms exhibited a steady decrease in crawling distances over the subsequent weeks while *mecr-1(RNAi)* worms remained more active throughout their life.

To confirm that depletion of MECR-1 affects mitochondrial function, an *hsp-6::gfp* mitochondrial heat shock protein reporter was used. Under mitochondrial stress conditions, *hsp-6* induces green fluorescent protein (GFP) expression [33]. Levels of *hsp-6::gfp* were analyzed in intact worms during chronic depletion of MECR-1 compared to control worms (Fig. 3D). At 7 d, *mecr-1(RNAi)* and control worms exhibited similar levels of *hsp-6::gfp* induction. However at 14 and 21 d, GFP expression was almost two-fold higher in *mecr-1(RNAi)* worms relative to controls, indicating that mitochondrial function was abnormal. Thus, MECR-1 was important for mitochondrial function and contributed to life span reduction in *C. elegans*.

## Discussion

Although all of the mammalian components of mitochondrial FASII have now been identified, there appears to be a (temporary) delay in generating FASII mouse knockouts. For example, human mitochondrial 2-*trans*-enoyl-thioester reductase MECR was exposed as early as 2003 [10] but, nevertheless, very recent work characterizing the deleterious effect of over-expressing Mecr on murine hearts was still restricted to heralding the initiation of knockout work [15]. If the efforts at generating mouse disruptions of the lipoic acid synthase gene *Lias* are in any way instructive to evaluating the chances for success at rapidly generating a FASII knockout mouse, then this does not bode well as these attempts have so far only led to embryonic lethality [34], precluding thorough investigations on live knockouts. For this reason, *C. elegans* could emerge as an important interim tool for developing our understanding of mitochondrial FASII in animals.

Here, the gene product of *C. elegans* W09H1.5 was identified as a 2-*trans*-enoyl-thioester reductase MECR-1 of mitochondrial FASII. By expressing MECR-1 in a yeast mutant devoid of native Etr1p, the nematode protein restored the mutant's missing functions to wild-type levels. *C. elegans* worms depleted of MECR-1 not only lived longer but were additionally more active and retained better tissue integrity in later life, revealing that their rate of aging was also decreased. This was commensurate with previous genome-wide RNAi results relating to W09H1.5 [28]. The finding presented here that MECR-1 is likely to be entrained in mitochondrial FASII provides a novel reference point from which to consider possible causes for why RNAi of *mecr-1* resulted in longer-lived and more agile nematodes.

As mentioned in the introduction, yeast mutants with a defective FASII present a number of mitochondrial anomalies, including underdeveloped organellar morphology, lack of assembled cytochrome complexes, an inability to synthesize lipoic acid, and in some cases a further loss of mitochondrial DNA [22]. In addition, these mutant yeast cells have altered mitochondrial RNA processing [14]. In *C. elegans* mitochondria, a lack of lipoic acid would primarily affect lipoylated enzymes such as the pyruvate dehydrogenase complex (PDH) and the related enzyme *α*-ketoglutarate dehydrogenase (*α*-KGDH) of the tricarboxylic acid (TCA) cycle, but also the glycine cleavage enzyme [13]. A defective PDH would foil the generation of mitochondrial acetyl-CoA from pyruvate, whereas a faulty *α*-KGDH will cause the TCA cycle to accumulate *α*-ketoglutarate and isocitrate. This will result in a metabolic shift since acetyl-CoA will now only come from the mitochondrial *β*-oxidation of those fatty acids originating in the bacterial lawn. This shift has been extensively studied [35], [36], and is accompanied by a flow of reduced electron carriers from glycolysis and *β*-oxidation, which will contribute to energy generation through the electron transfer chain, whereas isocitrate will enter the glyoxylate cycle to provide C_4_ building blocks for carbohydrate synthesis. Interestingly, interference with two other potential TCA cycle enzymes has been reported to extend life span in nematodes: subunits of NAD^+^-dependent isocitrate dehydrogenase represented by F43G9.1 and F35G12.2, and aconitase F54H12.1 [28], [37]. It may also be worth noting that the aforementioned metabolic shift towards the glyoxylate cycle has been shown previously to extend worm life span [35], [36].

As intimated above, a further way in which nematodes with depleted MECR-1 might be affected is represented by a defective mitochondrial tRNA processing machinery. Removal of tRNA sequences from these precursors involves two activities; i) a 3′-end endonucleolytic activity carried out by tRNase Z [38], and ii) a comparable activity at the 5′-end undertaken by a dedicated endonuclease RNase P [39]. RNase P expression is linked to mitochondrial FASII in both yeast [14] and vertebrates [11], and previous work demonstrated that a lack of any FASII enzyme decreased the efficiency of 5′ processing of mitochondrial precursor tRNAs by RNase P [14]. It follows that RNAi of *mecr-1* could affect indirectly the excision of mitochondrial tRNAs, which would be deleterious to the translation of mitochondrially encoded polypeptides and their assembly into the mitochondrial respiratory chain, akin to the situation found in yeast.

Clearly, it is not yet possible to explain the reason behind the increased longevity observed in *C. elegans* due to RNAi of *mecr-1*. However, a search of the literature and worm databases shows that RNAi of open reading frames with the potential to encode additional mitochondrial FASII participants, including candidates for mitochondrial acyl carrier protein (ACP), 3-oxoacyl-ACP reductase and 3-hydroxyacyl-ACP dehydratase, all result in life span extension [28], [40], [41]. Hence, although extensive studies will still be needed so as to complete the picture of mitochondrial FASII in nematodes, within the broader scope of addressing the requirement for this particular process in animal metabolism, *C. elegans* is likely to be a useful model.

## Materials and Methods

### Yeast strains, oligonucleotides and plasmid constructions

*S. cerevisiae* strains, plasmids, and oligonucleotides used are listed in Table 1. The *E. coli* strains DH10B and TOP 10 F' were used for plasmid amplifications and isolations. The wild-type yeast strain BJ1991 [42] and its *etr1*Δ derivative [6] were published previously. *URA3*-marked plasmids were introduced into BJ1991*etr1*Δ cells using a published protocol [43]. DNA manipulations and plasmid constructions were carried out following published methods [44]. The W09H1.5 gene was amplified from cDNA by polymerase chain reaction using the listed oligonucleotides (Table 1). The sequential steps of amplification, electrophoretic resolution, excision, purification and subsequent ligation of the amplicon to an *Eco*RV-digested pBluescript II plasmid vector (Stratagene, La Jolla, CA) are described [26]. The W09H1.5 insert was isolated from pBluescript following digestion with *Xba*I and *Xho*I restriction enzymes, and tethered to the *CTA1* promoter in a similarly digested pYE352:CTA1 [45] from which the *CTA1* open reading frame encoding yeast peroxisomal catalase A was removed. Nucleotides for a yeast MLS were not added. DNA sequencing of the insert encoding W09H1.5 verified that no mutations were introduced during the amplification process. Plasmid YEp352:ETR1 has been described previously [6].

**Table 1:** Strains, plasmids, and oligonucleotides used.

strain, plasmid, oligonucleotide	description	source of reference
*S. cerevisiae*		
1) BJ1991	*MATα leu2 ura3-52 trp1 pep4-3 prb1-122 gal2*	[42]
2) BJ1991*etr1*Δ^1a^	*ybr026c::kanMX*	[6]
yPLM37^2^	expressing yeast Cta1p from pPLM187	This study
yPLM38^2^	expressing nematode MECR-1 from pPLM62	This study
yPLM43^2^	expressing yeast Etr1p from pPLM188	This study
plasmid		
3) pBluescript II KS	pKS cloning vector	Stratagene
pPLM83^3^	pKS:W09H1.5 (*mecr-1*) in pBluescript	This study
4) YEp352	*URA3*-marked multicopy plasmid	[48]
5) pPLM187^44^	*CTA1* behind its own promoter (pYE352:CTA1)	[45]
pPLM188^5^	*ETR1* behind the *CTA1* promoter (pYE352:ETR1)	[6]
pPLM62^5^	*mecr-1* behind the *CTA1* promoter	This study
oligonucleotide		
Ce MECR1 5′ XbaI	TTATTCTAGATGTTAAAAGTTCTCAGTCTAC	This study
Ce MECR1 3′ XhoI	TATTCTCGAGTTATTCCAAAACGAAGAATTGC	This study

a The numbers in superscript following the strains' designation refer to their parental genotypes, e.g. BJ1991*etr1*Δ was derived from 1) BJ1991. The same principle applies to plasmids.

### Worm strains, maintenance and RNAi

*C. elegans* wild type N2 and SJ4100 [*zcIs13 hsp-6::gfp*] [33] worms were maintained on NGM plates seeded with OP50 bacteria at 16°C. RNAi was performed by feeding, as described previously. Briefly, bacteria were grown overnight at 37°C in LB+carb+tet then diluted 1∶5000 in LB+carb and grown for three h at 37°C. Double stranded RNA expression was induced with 3 mM IPTG for 15 min in culture at 37°C. Bacteria were then plated on NGM+carb+IPTG plates, grown overnight at room temperature, and stored at 4°C. The W09H1.5 feeding clone was obtained from MRC Gene Services (II- 8L09) and confirmed by sequencing. The control clone expressed L4440 empty vector. Gravid adult hermaphrodites were picked to NGM plates and allowed to crawl for ∼15 min to remove excess OP50 bacteria. About ten worms were transferred to RNAi plates and allowed to lay ∼40 eggs total. Adults were removed and RNAi plates were incubated at 16°C for the duration of the experiment. Any males were removed and not included in the analysis. Worms were transferred to fresh RNAi plates every week, or at higher frequencies (up to every day) as necessary to avoid contamination from the F2 generation during the peak egg laying period. Control and W09H1.5-depleted worms were counted and transferred with identical schedules.

### Longevity and aging assays

Times are relative to when eggs were laid, day 0. To calculate life span, worms were assessed every two days for movement (crawling, touch response, and pharyngeal pumping). If any movement was detectable, worms were considered alive. The log-rank test statistics for control versus *mecr-1(RNAi)* worms are as follows: chi-square  = 0.9, df  = 1, p = 0.338, indicating that the survival time was statistically different. The number of worms varied from test to test, and n refers to the number of replicates performed. Each longevity test contained 25–50 worms. Values are given as percentage alive due to the problem of worms escaping in the course of these experiments. In separate experiments, worm mobility, morphology, and mitochondrial dysfunction were measured on days 7, 14, 21, and 28 for five worms per analysis (new worms were used for each day). By day 28, the number of viable control worms was too small to obtain accurate measurements. Mobility was analyzed by transferring five worms to the bacterial lawn of individual NGM/OP50 plates, incubating the plates at room temperature for five min, removing the worms, and imaging the worm tracks using a Zeiss MZ Apo stereo microscope equipped with a SPOT camera. The magnification was kept constant throughout the experiment. Track length was calculated (in pixels) by tracing the axis of the tracks in Metamorph (Universal Imaging). For morphological and mitochondrial function analysis, images were performed at 10× magnification on a Zeiss Axioplan 2 microscope equipped with a CoolSnapfx cooled CCD and controlled by Metamorph. Morphology was analyzed in anesthetized (1% tricaine +0.1% tetramisole in 1X M9) worms. Worm length and width were measured in Metamorph. Mitochondrial stress was estimated from fluorescence intensity of worms expressing GFP under control of the mitochondrial heat shock factor *hsp-6* gene promoter. Anesthetized worms were imaged using an acquisition time of 200 msec, and the average GFP intensity of a 20-pixel diameter circle in the worm posterior was determined in Metamorph. Brood size was calculated by counting total eggs laid and hatched larvae from 10 individual worms throughout the egg-laying period. All values are given as mean ± s.e.m.

### Media and growth conditions

Standard yeast [46] and *E. coli* [47] media were processed as published. Solid rich-glucose YPD medium consisted of 1% (wt/vol) yeast extract - 2% (wt/vol) peptone (YP), 2% (wt/vol) D-glucose, and 2% (wt/vol) agar. YEp352-based episomal plasmids marked with the *URA3* gene [48] were maintained in transformed strains grown on synthetic defined SD-Ura medium consisting of 0.67% (wt/vol) yeast nitrogen base without amino acids, 2% (wt/vol) D-glucose, 3% (wt/vol) agar, with all supplements added except for uracil (Sigma-Aldrich Inc. MO USA). Synthetic complete SCglycerol medium was prepared essentially as described for SD-Ura, except that uracil was added and glucose was substituted with 3% (wt/vol) glycerol as the exclusive carbon source. Propagation of cells in triplicate cultures grown on YP-based oleic acid medium ahead of assays for 2-*trans*-enoyl-CoA reductase activity is described [31].

### Miscellaneous

Prior to the reductase activity assays, cells were ruptured using glass beads in an equal volume of breakage buffer made up of 50 mM KPi (pH 7.0), 200 mM KCl and 0.1% (wt/vol) Triton X-100. Protein concentrations were measured using the Bio-Rad protein assay (http://www.bio-rad.com/LifeScience/pdf/Bulletin_9004.pdf) according to the method of Bradford [49]. Enoyl reductase activity was determined spectrophotometrically at 23°C as described [50]. The reaction mixture was comprised of 50 mM KPi (pH 7.5) and 0.1 mg/ml bovine serum albumin, 125 µM NADPH, and 60 µM 2-*trans*-hexenoyl-CoA that was synthesized by way of the mixed anhydride system [51] as the substrate. Respiration competence was examined by overlaying cells spotted onto solid YPD medium with 0.1% (wt/vol) 2,3,5-triphenyltetrazolium chloride (TTC) in phosphate buffered saline and 1.5% (wt/vol) low-melting temperature agarose [52]. The content of lipoic acid in yeast strains was estimated using a biological assay relying on lipoic acid-deficient bacterial cells described previously [1], [53]. Mitochondrial localisation predictions were performed with MitoProt (mips.biochem.mpg.de/cgi-bin/proj/medgen/mitofilter), PSORT II (psort.nibb.ac.jp/form2. html), and TargetP (cbs.dtu.dk/services/TargetP/), which expose N-terminal mitochondrial targeting sequences [54], [55]. The homology study in Fig. 1 was generated with Multalin (npsa-pbil.ibcp.fr/cgi-bin/npsa_automat.pl?page=/NPSA/npsa_multalin.html) and Genedoc (www.nrbsc.org/gfx/genedoc/index.html).
